# The Use of Topical Lidocaine Versus Lidocaine Injection for Myringotomy and Ventilation Tube Insertion

**DOI:** 10.3390/medicina62030595

**Published:** 2026-03-21

**Authors:** Filip Bacan, Emili Dragaš, Mirta Peček, Iva Kelava, Andro Košec, Mihael Ries, Jakov Ajduk

**Affiliations:** 1 Department of Otorhinolaryngology and Head and Neck Surgery, University Hospital Centre Sestre Milosrdnice, 10000 Zagreb, Croatia; filipbacan@gmail.com (F.B.); ivkel@yahoo.com (I.K.); miskories@gmail.com (M.R.); jakov.ajduk@gmail.com (J.A.); 2 School of Medicine, University of Zagreb, Šalata 3, 10000 Zagreb, Croatia; emili.dragas1@gmail.com; 3 University Hospital for Tumors, University Hospital Centre Sestre Milosrdnice, 10000 Zagreb, Croatia; mirta.pec@gmail.com

**Keywords:** lidocaine, local anesthetic, lidocaine injection, myringotomy, otologic surgery, topical anesthetic, ventilation tube

## Abstract

*Background and Objectives*: Minor otologic procedures in adults are often performed under local anesthesia, either via injection or topical application, thereby avoiding general anesthesia-associated risks. This study aims to compare pain levels with the use of a lidocaine spray versus lidocaine injections. *Materials and Methods*: Fifty adult patients underwent local anesthetic myringotomy and ventilation tube placement, 30 unilaterally, and 20 bilaterally. Lidocaine injections were administered to 29 patients, and 21 received a lidocaine spray. Postoperatively, they were asked to mark their perceived pain level on a visual analogue scale (VAS, 0–100 mm), verbal rating scale (VRS, 0–3), and numeric rating scale (NRS, 0–10). Data normality was assessed using the Shapiro–Wilk test, continuous variables were analyzed using analysis of variance (ANOVA), and VRS outcomes were analyzed using binary logistic regression. A *p*-value ≤ 0.05 indicated statistical significance. *Results*: Pain levels were low in both groups, although consistently lower in the topical lidocaine group. The average VAS score was 23.14 mm (±14.69) for injection versus 9.76 mm (±11.41) for topical anesthesia (ANOVA, *p* = 0.002), while NRS scores averaged at 2.41 (±1.57) and 1.19 (±1.17), respectively (ANOVA, *p* = 0.009), indicating significantly lower pain with topical lidocaine. Logistic regression of the VRS indicated the same trend, although it did not reach statistical significance (OR = 0.153, 95% CI:0.017–1.389, *p* = 0.095). *Conclusions*: Lidocaine spray was associated with lower pain levels compared to lidocaine injections in patients undergoing myringotomy and ventilation tube placement. Our findings suggest that topical anesthesia may represent an effective alternative, offering a less invasive approach and reducing the needle-related psychological distress of patients.

## 1. Introduction

Minor ear procedures encompass myringotomies and ventilation tube placement, which are performed by creating an incision in the tympanic membrane, followed by placement of a ventilation tube to enable ventilation and continuous drainage of the tympanic cavity [1]. Its indications include chronic otitis media with effusion, recurrent acute otitis media, and suppurative collections in the middle ear [1]. It is frequently performed in the pediatric population as well as in adults, often as an office-based or outpatient procedure, requiring efficient and effective anesthetic techniques [2].

The use and development of local anesthetics in ear surgery began in 1884 with Emmanuel Zaufel using 10% cocaine in alcohol to anesthetize the eardrum [3]. Since then, numerous anesthetic methods have been developed and used [4]. Both general and local anesthesia are utilized, with general anesthesia typically reserved for pediatric patients and local anesthesia for adults, due to better cooperation and tolerance, particularly in outpatient settings [2]. The use of general anesthesia is associated with restlessness, disorientation, airway irritation, and emergence agitation, with most patients requiring additional analgesia due to postoperative pain [2,5]. Local anesthetics do not carry those associated risks while still providing adequate analgesia [2]. The ideal anesthetic should be painless to administer, have a rapid onset, and cause no tissue damage [6]. That is, however, not achievable with injectable anesthetic agents as they provide rapid and reliable analgesia but are painful to inject and lead to swelling of the ear canal, resulting in significant patient discomfort at the time of injection [7]. Topical anesthetics, therefore, provide a viable alternative [6]. Various agents are available in the form of creams, aqueous solutions, or sprays; however, creams are difficult to remove from the ear canal and take longer to achieve effectiveness [8]. Sprays, particularly lidocaine sprays, are commonly used as fast-acting agents in otorhinolaryngology for less invasive procedures in the larynx, throat, and nasal cavity [8]. Lidocaine exerts its anesthetic effects by binding to sodium channels reversibly, stopping sodium ions from moving into nerve fibers and causing depolarization [8]. Other, less commonly used local anesthetic agents include phenol, EMLA cream (a mixture of lidocaine and prilocaine), Bonain’s solution (cocaine hydrochloride, menthol, and phenol), and tetracaine injections [9].

Although lidocaine is frequently used in otolaryngology for providing local anesthesia, there is a relative scarcity of studies directly comparing the efficacy of topical lidocaine sprays with that of lidocaine injections in the management of procedural pain during minor ear surgeries. Both techniques are routinely applied in clinical practice, yet the choice between them is often based on clinician preference or institutional protocols rather than evidence-based guidelines. Understanding the relative effectiveness, onset of action, and patient-reported pain levels associated with these two approaches is crucial for optimizing patient comfort, especially in procedures such as myringotomy with ventilation tube placement.

Therefore, the primary objective of our study was to systematically compare the analgesic efficacy of topical lidocaine spray versus lidocaine injection in patients undergoing myringotomy with ventilation tube placement. By evaluating pain using standardized scales postoperatively, we aimed to provide evidence-based guidance to clinicians regarding the most effective method of local anesthesia for myringotomy and ventilation tube insertion, potentially improving patient satisfaction and procedural outcomes.

## 2. Materials and Methods

This cross-sectional observational study was designed to compare the anesthetic effect of topical lidocaine and lidocaine injection. The cross-sectional design was chosen because it allows for a precise snapshot of pain associated with different local anesthesia techniques. By measuring pain at a single standardized time point, variability due to healing or delayed pain perception is minimized.

Patients who underwent a myringotomy with ventilation tube placement were recruited at a tertiary referral otology center throughout 2025 and 2026, following the Institutional Review Board (IRB) approval. Data were collected at a single time using standardized questionnaires.

The study was designed according to the Strengthening the Reporting of Observational Studies in Epidemiology criteria (STROBE) and received institutional ethics review board approval (IRB). Written informed consent was obtained from all participants prior to their participation in the study.

The inclusion criteria were patients who required myringotomy, either unilaterally or bilaterally, with ventilation tube placement. The procedure had to be performed with local anesthesia, either in the form of injection or spray. The exclusion criteria were patients who required general anesthesia to perform the procedure, either due to their age or other factors.

**Figure 1:**
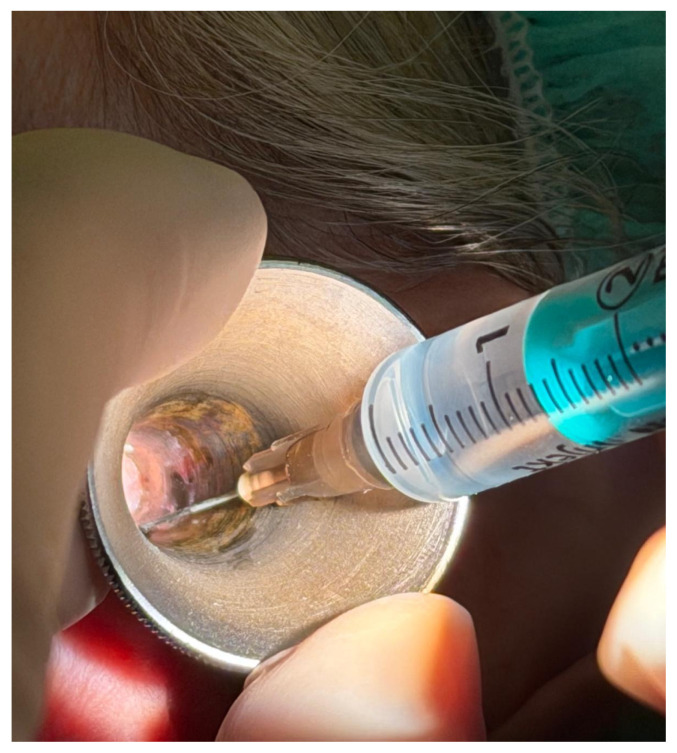
The lidocaine injection technique used in our study. The solution is injected using a four-quadrant injection technique in the external auditory canal through the ear speculum.

**Figure 2:**
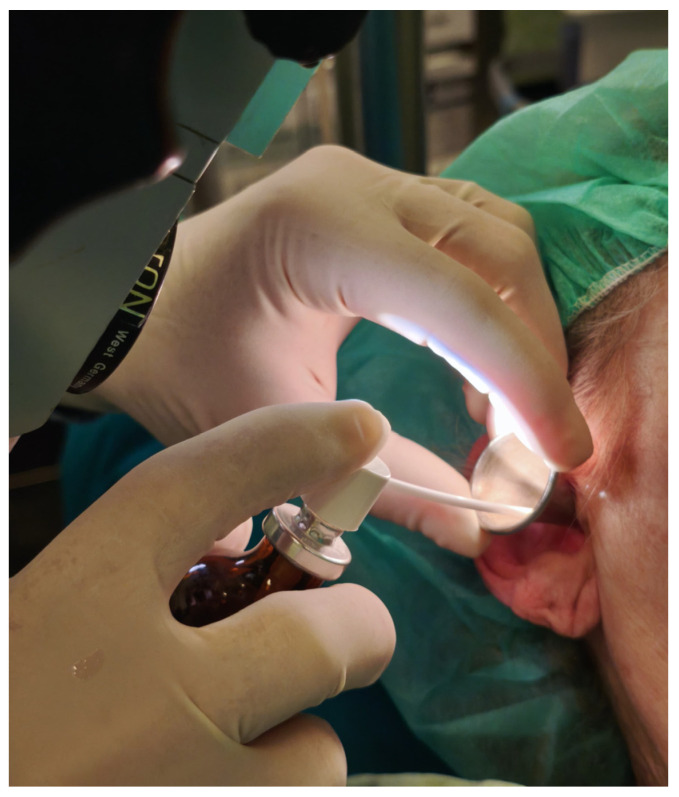
The application of the lidocaine spray. Through the ear speculum, four sprays are applied in each ear.

Local anesthesia was achieved either using a lidocaine injection or a lidocaine spray. The concentration and administration technique for both injection and spray were chosen to ensure adequate anesthesia while minimizing systemic absorption. The injection technique utilized a 20 mg/mL lidocaine chloride solution mixed with 1:200,000 adrenaline (Belupo, Ulica Danica 5, 48000, Koprivnica, Croatia), which is injected using a four-quadrant injection technique in the external auditory canal to achieve a complete nerve block of the area (Figure 1). The addition of epinephrine is used to reduce local bleeding. The spray used was a 100 mg/mL lidocaine spray (Belupo, Ulica Danica 5, 48000, Koprivnica, Croatia), and four sprays (0.1 mL each) were applied in each ear, ensuring adequate distribution throughout the ear canal (Figure 2). Both the spray and injection were administered through an ear speculum, as visible in Figure 1 and Figure 2.

After the procedure, in the postoperative period, patients were asked to complete a short questionnaire. The questionnaire consisted of a visual analog scale (VAS), verbal rating scale (VRS), and numeric rating scale (NRS). Using three complementary pain scales allowed for a robust assessment of the subjective experience of pain. The VAS provides a continuous measure, sensitive to small differences in pain perception, while the VRS offers categorical grading that is easily interpretable by patients. The NRS allows for rapid assessment and has been validated in various clinical settings. A sample power analysis was made based on the projected average pain level difference being one VAS point (0–10). Alpha was set at 0.05, and the power of the test was 80%. Power analysis set the minimum required sample size to establish statistical significance at 16 subjects in every patient group, 32 total. Data analysis was aimed at testing the impact of anesthesia types on pain levels in patients undergoing myringotomy and ventilation tube insertion. Clinical covariates were patient age in years, gender (male/female), and laterality of the procedure (unilateral versus bilateral), while the dependent predictor variable was the type of anesthesia (topical or infiltration). Outcome variables were: (1) pain levels measured in millimeters (mm) on a VAS of 100 mm, (2) pain levels on a 4-point Likert VRS, with patients classifying the pain from 0 denoting no pain, 1 denoting mild pain, 2 denoting intermediate pain, 3 denoting intense pain, and (3) a numeric rating scale (NRS) measuring 0–10, with 0 denoting no pain, and 10 the worst pain imaginable. When surgical laterality is mentioned, it refers to whether the procedure was done on one or both ears (unilateral or bilateral).

The analysis of data normality distribution was tested with the Shapiro–Wilk test, a sensitive method for small to moderate sample sizes, and according to the results, parametric tests were used, as well as the corresponding display of continuous values (arithmetic mean and standard deviation). VAS scores were analyzed using binary logistic regression. VAS and NRS, treated as continuous variables, were analyzed using ANOVA. All statistical tests were two-tailed. Values of *p* less than or equal to 0.05 are determined to be statistically significant. Statistical data processing was performed using the software JASP, version 0.96.0.

## 3. Results

This study included 50 patients who underwent myringotomy with ventilation tube placements. The average age of patients was 51 years. Among them, there were 19 men and 31 women. A one-sided procedure was performed in 30 patients, while 20 patients underwent a bilateral procedure. Lidocaine was injected in 29 patients, while 21 received the lidocaine spray. These baseline characteristics are presented in Table 1.

Of the total number of patients, 27 patients reported mild pain, 9 patients reported moderate pain, and 14 patients reported no pain at VAS. Considering VRS, 24 patients reported moderate pain, 24 reported no pain at all, and 2 reported intermediate pain. The median level of pain for both procedures on NRS was 1.9. Statistical tests were performed to see whether the difference between the two groups was statistically significant in all measurements.

The results are further presented based on the pain level test used as an assessment tool for pain in patients.

### 3.1. VAS Scores

The mean VAS score was 23.14 mm (±14.69) in patients receiving lidocaine injections and 9.76 mm (±11.41) in those receiving the lidocaine spray. On the performed ANOVA test, where both anesthesia type and laterality of the procedure were considered, there was a statistically significant difference between these two groups, with lower pain levels observed in the group receiving the lidocaine spray (*p* = 0.002) (Table 2). The main effect of surgical laterality was not statistically significant (*p* = 0.969), and no significant interaction between anesthesia type and laterality was observed (*p* = 0.999) (Table 2).

These results indicate that while topical anesthesia was associated with lower VAS scores, the laterality of the procedure did not substantially influence pain perception.

Additionally, we compared VAS results separately in patients undergoing a unilateral and a bilateral procedure. There was a statistically significant lower pain level reported on the VAS in patients undergoing a unilateral procedure with topical anesthesia compared to a lidocaine injection (*p* = 0.026) (Table 3). The same was found in patients undergoing a bilateral procedure (*p* = 0.032) (Table 4).

### 3.2. VRS Scores

The four-point VRS was divided into two categories: mild pain (scores 0 and 1), and severe pain (scores 2 and 3). Mild pain was reported by 41 patients, of whom 22 received the lidocaine injection, and 9 reported severe pain, 8 of whom received the lidocaine injection.

Binary logistic regression including both the anesthesia type and the laterality of the procedure did not reach statistical significance (overall model, *p* = 0.068). Topical anesthesia showed a trend toward lower odds of severe pain compared with injectable anesthesia, indicating a potential but not statistically significant clinical benefit in this cohort (OR = 0.153, 95% CI: 0.017–1.389, *p* = 0.095).

Additionally, we analyzed the VRS scores between anesthesia types in patients undergoing a unilateral and a bilateral procedure separately. There were no statistically significant differences in VRS scores between the anesthesia types in patients undergoing a unilateral procedure (*p* = 0.275). However, in patients undergoing a bilateral procedure, a statistically significant difference was found (*p* = 0.045).

### 3.3. NRS Scores

The mean NRS score was 2.41 (±1.57) in patients receiving the lidocaine injection and 1.19 (±1.17) in patients receiving the lidocaine spray. The ANOVA test demonstrated statistically significantly lower pain levels in patients receiving the topical anesthetic, when both anesthesia type and the laterality of the procedure were considered (*p* = 0.009) (Table 5). The main effect of surgical laterality was not statistically significant (*p* = 0.730), and there was no significant interaction between anesthesia type and laterality (*p* = 0.823) (Table 5).

These findings are consistent with the VAS results, indicating that topical anesthesia was associated with lower pain scores, while laterality had no detectable influence in this cohort.

Additionally, we compared NRS results separately in patients undergoing a unilateral and a bilateral procedure. There was not a statistically significant difference between anesthesia types in patients undergoing a unilateral procedure (*p* = 0.069) (Table 6). A similar result was found in patients undergoing a bilateral procedure, which, as well, did not reach statistical significance (*p* = 0.063) (Table 7).

## 4. Discussion

In our study, we compared pain levels in myringotomy and ventilation tube placement anesthetized with a lidocaine spray compared with a lidocaine injection by using standardized pain level scales (VAS, VRS, NRS). Statistically significant lower pain levels were reported by VAS and NRS in the group receiving topical lidocaine compared to patients receiving lidocaine injections. Additionally, patients were stratified based on the laterality of the procedure, but no significant interaction was found between anesthesia type and laterality. When pain was assessed by VRS considering both anesthesia type and laterality, the result followed the trend of other assessment tools but was not considered statistically significant (*p* = 0.068). When pain levels were compared between patients undergoing a unilateral and a bilateral procedure, only VRS scores differed, with patients undergoing a bilateral procedure reporting significantly lower pain levels with topical lidocaine.

Pain is inherently a subjective experience and cannot be measured directly but can be assessed by standardized pain scales. Previous studies have examined the efficacy of various topical anesthetics in minor procedures. For example, Nichani et al. reported that two different topical anesthetics, tetracaine and EMLA, were equally effective in reducing pain [10]. To the best of our knowledge, no previous study has directly compared pain levels between lidocaine administered via injection and via spray in minor ear procedures. In our study, we used a VAS to assess pain intensity, with most patients reporting mild pain (54%), while 28% reported no pain at all. Similar findings were reported by McNally et Izzat [5]. To further quantify pain, we also employed two additional pain scales, VRS and NRS, both of which confirmed overall low pain levels, with lower scores reported by patients receiving topical anesthetics.

Considering the administration process, the result is somewhat expected. Injectable lidocaine, although fast acting, is accompanied by pain in the application process and swelling of the external ear canal, causing discomfort in patients, with some studies describing this pain as similar to that of performing a myringotomy without anesthesia altogether [7]. A study by Caddick et al. demonstrated that a significant amount of anxiety during awake operations is a result of the injection of a local anesthetic [11]. The paper by Roberts et al. concluded that topical anesthetics, in their case the EMLA cream, provided the same level of anesthesia as the injections, with overall less discomfort [7]. However, lidocaine injections are still a widely used technique due to their efficiency and reliability [7].

On the other hand, topical lidocaine was associated with lower pain levels in patients. In our study, we chose a lidocaine spray formulation, as did McNally et al. [8]. Although their study did not compare the spray with injections, they reported overall low pain levels following the use of the spray, consistent with our findings [8]. A formulation of lidocaine with phenylephrine is also mentioned in the literature, with authors reporting satisfaction with the spray formulation [12].

Along with sprays, creams are most often mentioned in the literature, with both formulations possessing distinct properties that can affect overall performance. Topical anesthetic in the form of an EMLA cream (eutectic mixture of local anesthetics—lidocaine and prilocaine) is reported as an effective method for anesthesia of the tympanic membrane and is often mentioned in the literature [13,14]. It is, however, accompanied by certain setbacks, as it has to be administered at least 30 min before surgery, and the formation of air bubbles may result in incomplete anesthesia of the tympanic membrane [7,14]. The spray formulation, on the other hand, is shown to guarantee a thorough covering of the tympanic membrane, creating an even analgesic effect, similar to that of an injection [8]. Additionally, it aids in the elimination of wax from the ear canal by softening it, and does not require a microscope to administer, making it easier to use in outpatient settings [8].

In our study, we used a lidocaine spray as the anesthetic of choice, even though other options have been described in the literature, such as a phenol aqueous solution. It results in a chemical burn of partial thickness, necrosis of sensitive nerve ends, and immediate anesthesia [15]. This creates a potential risk of perforation of the tympanic membrane; however, this risk has been described as very low in the literature [15,16,17].

As aqueous local anesthetic treatments are usually poorly absorbed through the skin, one of our concerns with the lidocaine spray was its potential ineffectiveness. This was, however, already disproved in the literature and further solidified by our results, with a potential explanation being the thin epidermal layer of the tympanic membrane [16].

Another consideration that contributed to our decision to choose a spray as a topical anesthetic is that, in contrast to a cream, which may still leave behind residue despite extensive microsuction and consequently obstruct an already constrained view of the tympanic membrane, the excess aqueous solution may be easily removed. The residual contents could raise concern for postoperative complications such as vertigo or facial palsy, as was reported by Hoffman and Li in their retrospective analysis of the use of 8% tetracaine solution for a myringotomy and tube insertion, where these complications were reported in patients receiving postoperative otobiotic drops, possibly carrying the tetracaine in the tympanic cavity [18]. Similar symptoms, the main being vertigo, are experienced after intratympanic insertion of lidocaine [19]. A study published in 2019 found that the use of topical anesthetics in myringotomy and ventilation tube insertion does not increase the risk of sensorineural hearing loss, although this risk of residual transfer should still be considered when choosing a topical over an injectable local anesthetic [20]. It is often the reason why clinicians refrain from using topical anesthetics in these procedures.

Although there is not an ideal local anesthetic agent, the results of this paper might encourage the use of lidocaine sprays in minor ear procedures compared with lidocaine injections. Its additional benefit lies in the potential decrease in patient anxiety associated with injection, and should be clearly communicated to patients to further decrease anxiety levels [11]. It is important to note that the choice of an anesthetic should understandably be tailored to the patient, but also to the surgeon’s preferences, while taking into consideration the clinical environment, operational capacity, and resource availability.

Another factor in our analysis was the laterality of the procedure. As a proportion of patients received a bilateral procedure, precisely 40% of patients, it could be assumed that their pain levels would be higher and potentially influence the observed effect of anesthesia type on pain. However, when pain was assessed using VAS and NRS, two-way ANOVA did not demonstrate a significant interaction between anesthesia type and surgical laterality. Furthermore, surgical laterality was not significantly associated with pain scores. These findings suggest that the laterality of the procedure did not substantially influence pain perception in our cohort. Similarly, in the logistic regression analysis of VRS categories, neither anesthesia type nor surgical laterality showed a statistically significant association with the occurrence of severe pain, although topical anesthesia demonstrated a tendency toward lower odds of severe pain compared with injectable anesthesia.

Additionally, we performed a stratified analysis according to procedure laterality, separately for unilateral and bilateral cases. When pain was assessed using VAS, topical lidocaine was associated with significantly lower pain levels in both groups. In contrast, no statistically significant differences between anesthesia types based on the laterality of the procedure were observed using NRS. However, with VRS, a statistically significant difference in favor of topical lidocaine was found in patients undergoing bilateral procedures, whereas no significant difference was observed in unilateral cases. Therefore, due to the inconsistency of these results, it is not possible to conclude that either topical lidocaine or injectable lidocaine would be preferential when performing either a unilateral or a bilateral myringotomy with tube insertion.

Certain limitations to this study should be acknowledged. The main limitation is the small sample size; therefore, further research in a larger number of patients is needed to further establish these differences. Additionally, patient selection was limited to the adult population. Regarding the injection of local anesthesia, the application itself may vary significantly between physicians, precisely by the number of needle punctures of the skin, which can influence the reported pain level. These variations could also be a result of the unique local anatomy of each patient, making it difficult to fully standardize the application technique. As mentioned before, pain is a subjective experience, and it, therefore, can vary significantly between patients due to their individual susceptibility to pain. We did use standardized pain level scales, but this should still be considered in the interpretation.

## 5. Conclusions

Our study aimed to analyze the difference in reported pain levels among patients undergoing myringotomy and ventilation tube insertion anesthetized with a lidocaine spray compared to a lidocaine injection. Both groups experienced generally low levels of pain; however, patients who received the lidocaine spray reported lower pain scores compared with those who received lidocaine injections across all measured outcomes. These results indicate that topical anesthesia could be an effective alternative, providing a less invasive option while also minimizing the psychological discomfort associated with injections.

## Figures and Tables

**Table 1:** Baseline characteristics of the included patients and procedures performed.

Patient Characteristics	Value
Number of patients (N)	50
Age, mean (years)	51
Male (N)	19 (38%)
Female (N)	31 (62%)
Procedure Characteristics	Value
One-sided procedure (N)	30 (60%)
Two-sided procedure (N)	20 (40%)
Lidocaine injection (N)	29 (58%)
Lidocaine spray (N)	21 (42%)

**Table 2:** The ANOVA test for VAS scores showed a statistically significant difference between the topical and infiltration anesthesia (*p* = 0.002), while no statistical significance was found between anesthesia types and the laterality of the procedure (*p* = 0.999).

Cases	Sum of Squares	df	Mean Square	F	*p*
Anesthesia Type	1963.330	1	1963.330	10.442	0.002
Laterality	0.281	1	0.281	0.001	0.969
Anesthesia type × Laterality	3.861 × 10^−4^	1	3.861 × 10^−4^	2.053 × 10^−6^	0.999

Note. Type III Sum of Squares.

**Table 3:** The ANOVA test for VAS scores in patients undergoing a unilateral procedure showed a statistically significant difference between the types of anesthesia (*p* = 0.026).

Cases	Sum of Squares	df	Mean Square	F	*p*
Anesthesia Type	1136	1	1136.1	5.500	0.026

**Table 4:** The ANOVA test for VAS scores in patients undergoing a bilateral procedure showed a statistically significant difference between the types of anesthesia (*p* = 0.032).

Cases	Sum of Squares	df	Mean Square	F	*p*
Anesthesia Type	864.0	1	864.0	5.428	0.032

**Table 5:** The ANOVA test of NRS scores indicates lower pain levels in patients receiving topical lidocaine (*p* = 0.009). No statistically significant interaction between anesthesia type and the laterality of the procedure was observed (*p* = 0.823).

Cases	Sum of Squares	df	Mean Square	F	*p*
Anesthesia Type	15.521	1	15.521	7.442	0.009
Laterality	0.251	1	0.251	0.120	0.730
Anesthesia type × Laterality	0.105	1	0.105	0.050	0.823

Note. Type III Sum of Squares.

**Table 6:** The ANOVA test of NRS scores in patients undergoing a unilateral procedure does not show a statistically significant difference between anesthesia types (*p* = 0.069).

Cases	Sum of Squares	df	Mean Square	F	*p*
Anesthesia Type	7.557	1	7.557	3.578	0.069

**Table 7:** The ANOVA test of NRS scores in patients undergoing a bilateral procedure does not show a statistically significant difference between anesthesia types (*p* = 0.063).

Cases	Sum of Squares	df	Mean Square	F	*p*
Anesthesia Type	8.008	1	8.008	3.918	0.063

## Data Availability

Data available from the corresponding author upon reasonable request.

## Abbreviations

The following abbreviations are used in this manuscript:

EMLA	Eutectic Mixture of Local Anesthetics
VAS	Visal analog scale
VRS	Verbal rating scale
NRS	Numeric rating scale
ANOVA	Analysis of variance
